# Influence of Nitrogen Source on 2,4-diacetylphloroglucinol Production by the Biocontrol Strain Pf-5

**DOI:** 10.2174/1874285800802010074

**Published:** 2008-05-30

**Authors:** Hultberg M, Alsanius B

**Affiliations:** Department of Horticulture, Microbial Horticultural Laboratory, Swedish University of Agricultural Sciences, Alnarp, Sweden

**Keywords:** Biological control, 2, 4-diacetylphloroglucinol, greenhouse, nitrogen source, Pseudomonas fluorescens Pf-5

## Abstract

The production of 2,4-diacetylphloroglucinol (DAPG) by the biocontrol agent *Pseudomonas fluorescens *Pf-5 was studied in nutrient-solution based media with varying nitrogen content. No production of DAPG was observed when organic nitrogen was omitted from the media, regardless of the inorganic nitrogen source used. Furthermore, a micromolar concentration range of organic nitrogen was insufficient to sustain production. When a millimolar concentration range of organic nitrogen was used, DAPG production was observed in the medium with ammonium as the inorganic nitrogen source. No production was observed in the treatments with ammonium and nitrate or nitrate only, despite growth of the bacterial strain being the same for all treatments. These results suggest that it is possible to manipulate the nutrient solution to increase the reliability and efficacy of biological control agents.

## INTRODUCTION

Some groups of pseudomonads, indigenous in the rhizosphere, produce and excrete secondary metabolites that are inhibitory to plant pathogens [1]. They offer a sustainable alternative to pesticides and can provide disease control when the use of pesticides is legally restricted. Among the metabolites produced by *Pseudomonas* spp., the compound 2,4-diacetylphloroglucinol (DAPG) has been intensively studied due to its broad spectrum activity towards the commercially important plant pathogens* Gaeumannomyces graminis* var. *tritici*, *Thielaviopsis basicola*, *Pythium ultimum* and *Rhizoctonia solani* [2]. DAPG-producing pseudomonads have been detected in the rhizosphere of various crops, e.g. cucumber, maize, pea, tobacco, tomato, wheat and oat [3-9]. The presence of the compound has been confirmed in different cropping systems such as soils and hydroponic systems [10, 11] and the importance of DAPG *in situ* has been verified with DAPG- deficient mutants [12].

An obstacle in the development of efficient biocontrol agents is the inconsistency of their performance [13]. The complex *in situ* condition in the natural rhizosphere influences survival, growth and production of secondary metabolites by the biocontrol strains. With respect to DAPG production, mineral and carbon sources as well as metabolites released by indigenous microflora and plants have been identified as being critical factors [12, 14-16].

An important measure in developing reliable biocontrol systems is to identify suitable conditions for optimal metabolite formation by the biocontrol strains and to provide these conditions in plant cultivation systems. For outdoor field cropping systems this is a considerable challenge due to unavoidable variations in environmental conditions. However, greenhouse cropping systems with their more controlled environment offer a niche for successful development of biocontrol [17]. However, it is still not an easy task bearing in mind the multifactorial nature of biocontrol interactions and given that the needs of the plant are the main concern.

The present study focuses on the potential to improve the reliability of biological control in soilless greenhouse systems. The objective was to determine the impact of nitrogen source on DAPG production. The experiment was performed *in vitro*, but with hydroponic cultivation of tomato in mind. The well-known biocontrol strain Pf-5 [18] was cultivated in media based on the nutrient solution commonly used in tomato hydroponic systems [19]. The ratio of ammonium to nitrate ions was varied and the media were amended with organic nitrogen sources found in tomato root exudates [20], and the bacterial growth and DAPG production were studied.

## MATERIALS AND METHODS

### Microorganism and Inoculum Preparation

The bacterial strain Pf-5 [18] was pre-cultured on nutrient agar for 48 hours at room temperature. This strain was kindly provided by Dr. Loper, University of California, USA, and stock cultures of the strain were kept in 40% glycerol (v/v) at -80 °C. A single colony from the nutrient agar was transferred to 15 mL of nutrient broth (Difco® 0003-17) in a 100 mL Erlenmeyer flask and incubated on a rotary shaker (180 rpm, 24 hours at room temperature). The cells were centrifuged (8000 g, 4 °C, 15 minutes; Avanti J-20, Beckman Coulter, CA, USA). The supernatant was discarded and the pellet was washed once in 0.85% NaCl and finally dissolved in 0.85% NaCl to an optical density of 1.0 at 620 nm.

### Composition of the Media and Culture Conditions

In medium A no organic nitrogen was added, in medium B organic nitrogen was added in a millimolar concentration range and in medium C organic nitrogen was added in a micromolar concentration range. In treatment 1 (A1, B1 and C1) the inorganic nitrogen was provided as both nitrate and ammonium nitrate. In treatment 2 (A2, B2 and C2) the inorganic nitrogen was provided as nitrate only and in treatment 3 (A3, B3 and C3) the inorganic nitrogen was provided as ammonium only. The total concentration of inorganic nitrogen was 17 mM in all media. Glucose (Sigma-Aldrich Co., USA) (2%) was added as a carbon source in all media.

The composition of salts and pH of medium A1 corresponded to those in the nutrient solution commonly used for hydroponic cultivation of tomato: KNO_3_ 8.0, NH_4_NO_3_ 0.6, MgSO_4_ 1.9, KH_2_PO_4 _1.5, Ca(NO_3_)_2_ 4.2 **(mM)**, Fe-EDTA 18.7, MnSO_4_ 12.5, ZnSO_4_ 6.3, H_3_BO_3_ 31.3, CuCl_2_ 0.94, Na_2_MoO_4_ 0.6 **(μM)**, pH 5.9. Medium A2 contained nitrate as the only nitrogen source and NH_4_NO_3 _was excluded and replaced with an equal amount of KNO_3_ and Ca(NO_3_)_2_. Medium A3 contained ammonium (NH_4_Cl) as the sole nitrogen source.

In media B1, B2 and B3, the composition of the broth was the same as described above and the amino acids aspartic acid, glutamic acid, isoleucine, leucine and lysine were added at concentrations of 7.5, 6.8, 7.6, 7.6 and 6.8 mM respectively. These amino acids have previously been shown to be present in root exudates from tomato [20]. All amino acids were L-form and purchased from Sigma-Aldrich Co., USA.

In media C1, C2 and C3, the composition of the broth was the same as described for treatment B1, B2 and B3, except that the concentration of the amino acids was at micromolar level (concentrations of 7.5, 6.8, 7.6, 7.6 and 6.8 μM respectively). These concentrations are approximately in agreement with the concentrations observed in tomato root exudates [20].

All media were supplemented with adenine, cytosine, guanine, uracil, thymine and vitamins according to Slininger and Shea-Andersh [21]. All salts, amino acids and vitamins were added to the broth after it had been autoclaved and cooled down. After this the pH was set to 5.9 with NaOH and the broth was membrane-filtered (0.22 μm, Sarstedt, Germany) before the start of the experiment.

Nutrient broth amended with 2% glucose (NBglu) has previously been shown to be superior for DAPG production by strain Pf-5 [22] and was included as a control.

A volume of 15 mL of the respective medium was added to 100 mL Erlenmeyer flasks and an aliquot of 100 μL inoculum was added to each flask. The flasks were incubated on a rotary shaker (180 rpm at room temperature) and samples were withdrawn after 24, 48 and 72 hours.

### Analyses

DAPG was analysed according to Slininger and Shea-Andersh [21] with slight modifications. Samples were taken after 24, 48 and 72 hours of incubation and centrifuged (8000 g at 4 °C for 15 minutes). A volume of 20 μL of the supernatant was applied to a Waters Symmetry C-18 column (WAT054275, 5 μm particle size, 4.6 diam., 250 mm long) and a mobile phase of 65/35 acetonitrile (HPLC grade)/water with 0.1% glacial acetic acid with UV detection at 270 nm, flow 1 mL min^-1^. Concentrations were calculated relative to standards prepared using DAPG (Toronto Research Chemicals Inc., Ontario, Canada, lot: 1-JS-36-1), retention time 5.8 min. The detection limit for DAPG was approximately 0.5 μg mL^-1^. The spectrum of the peak was compared to the standard and published spectrum [23] to ensure the identity of the peak.

The optical density at 620 nm was monitored over time and the generation time in the various media was calculated. Protein content in the samples was measured after 72 hours of growth. An aliquot of 3 mL of cell suspension was centrifuged (8000 g at 4 °C for 15 minutes). The pellet was washed twice and then re-suspended in sterile water. The detergent Triton X (Sigma-Aldrich Co., USA) was added to the sample at a concentration of 0.1% (v/v) and the samples were intensively vortexed. Thereafter, the samples were frozen for 24 hours and after thawing they were homogenised using a glass homogeniser (Duall® 21, Kontes Glass Co., Vineland, NJ, USA). The protein content in each sample was determined by the method of Lowry *et al.* [24], using a standard curve prepared with bovine serum albumin (Sigma-Aldrich Co., USA).

### Experimental set-Up and Statistics

All experiments were performed with four replicates and repeated once. Data were analysed by analysis of variance followed by Tukey´s multiple comparison test and P<0.05 was considered significant (Minitab, version 14).

## RESULTS

**Fig. (1). Production of DAPG over time by the strain Pf-5.**
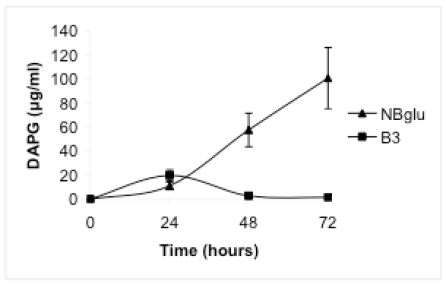
Production of DAPG by strain Pf-5 during a 72-hour period in the media NBglu and B3. Values represent the mean of four replicates. Error bars are provided if the standard error of the mean exceeds symbol dimensions.

When Pf-5 was grown in NBglu, a high production of DAPG was observed. After 24 hours the concentration in the medium was 11.0 μg mL^-1^ and the concentration increased during the following 48 hours to 100.7 μg mL^-1^ (Fig. **1**). The generation time was 6.1 hours and the optical density after 72 hours was 1.54, corresponding to 2926 μg mL^-1^ of protein (Table **1**).

In medium A amended with only inorganic nitrogen sources, nitrate and ammonium nitrate (A1), nitrate only (A2) and ammonium only (A3), no production of DAPG was observed (Table **1**). No significant differences were observed between the various nitrogen sources as regards bacterial growth. The generation time, optical density and protein content are presented in Table **1**.

**Fig. (2). Growth over time by the strain Pf-5.**
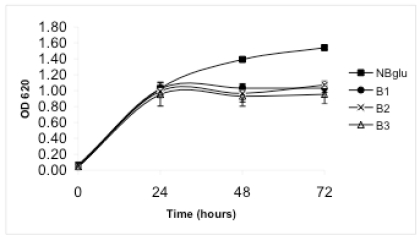
Optical density over time when the strain Pf-5 was grown in the media NBglu, B1, B2 and B3. Values represent the mean of four replicates. Error bars are provided if the standard error of the mean exceeds symbol dimensions.

When amino acids were added in a millimolar concentration range (treatment B), DAPG production was observed in medium B3, in which ammonium was used as the inorganic nitrogen source. After 24 hours the concentration was 19.8 µg mL^-1^ and declined thereafter (Fig. **1**). Only low DAPG production, below the detection limit, was observed in some samples from medium B1 or B2. Treatment B supported considerably higher bacterial growth than treatments A and C, but the bacterial growth was still significantly lower than in NBglu (Table **1**, Fig. **2**). No significant differences were observed when the optical density after 72 hours was compared within treatment B (Table **1**, Fig. **2**). However, the protein content was significantly higher for treatment B2 compared with treatments B1 and B3 (Table **1**).

When amino acids were added to a micromolar concentration range (treatment C) no DAPG production was observed. The bacterial growth was low overall and not significantly different from treatment A (Table **1**).

## DISCUSSION

The main finding of this *in vitro* study was that the nitrogen source, both inorganic and organic, clearly influences DAPG production by biocontrol strain Pf-5. Screenings for DAPG production *in vitro* have mostly been done using complex bacterial growth media or using a defined media and addition of yeast extract or casamino acids [3, 10, 15,16, 21, 25]. In the present study no organic nitrogen was added in treatment A. This resulted in poor growth and no production of DAPG, independent of the inorganic nitrogen source. These results are in contrast to results obtained by Siddiqui and Shaukat [26], who observed a nematicidal activity of filtrate from the DAPG-producing strain CHA0 when cultured on a medium lacking organic nitrogen. However, in their study it was not confirmed that the nematicidal activity was due to DAPG and other metabolites might have caused the biocontrol effect.

When the medium in the present study was supplemented with organic nitrogen in a millimolar concentration range, a significantly higher growth rate was observed. However, substantial DAPG production was only observed in medium B3, in which ammonium was included as the inorganic nitrogen source. This result cannot be explained by an increased cellular density in this treatment, as there was no difference in optical density within treatment B. Furthermore, expressing cellular growth as protein yield showed that medium B2 sustained the highest growth despite the fact that DAPG production was only occasionally observed in this medium and then at concentrations below 0.5 µg mL^-1^.

The result concerning an increased production of DAPG when ammonium was included as the inorganic nitrogen source is in agreement with other results. Crowley *et al*. [27] observed that DAPG production by strain *Pseudomonas fluorescens* F113 was stimulated when the nitrogen source was in form of ammonium ions. Duffy and Defago [15] also reported an increased production of DAPG by the strain *Pseudomonas fluorescens* CHA0 when ammonium was added. However, the question concerning whether ammonium ions stimulate DAPG production in general or only in selected strains needs to be further investigated. It has previously been shown that the carbon source that stimulates the highest DAPG production is strain-specific [15] and it is possible that a similar situation may apply regarding the inorganic nitrogen source.

Previous work has shown that the amino acid concentration in tomato root exudate is in the micromolar concentration range [20]. The results of the present study show that this concentration is to low to support production of DAPG *in vitro*. However, DAPG has been detected in tomato hydroponic systems [11]. In these plant cultivation systems, especially if recirculated, organic nitrogen is accumulated and it is possible that sufficient levels are reached. It is also likely that nutrient-rich microsites in the immediate surroundings of the root provide a niche. Furthermore, microbial products have been suggested to trigger amino acid exudation from roots [28]. Plants naturally cycle amino acids across their root cell plasma membranes and Phillips *et al*. [28] showed that in the presence of DAPG, the amino acid uptake by the plant was blocked. The previously cited study by Simons *et al*. [20], which showed a micromolar concentration range of amino acids in tomato root exudates, was performed on axenically cultured tomato roots. It is possible that higher levels of amino acid exudation would have been observed if those experiments had been performed in the presence of DAPG.

The findings of the present study suggest that it would be possible to adapt hydroponic cultivation systems to increase the production of DAPG by added biocontrol strains. The preferred inorganic nitrogen source should be ammonium and the amount of organic carbon should not be below a millimolar concentration range. However, the needs of plants also have to be addressed and possible effects of these changes on plants need to be further investigated.

## Figures and Tables

**Table 1. Growth and Production of DAPG by the Strain Pf-5.** DAPG production, generation time, optical density and protein content when the strain Pf-5 was grown in media with varying nitro-gen content. In treatment A only inorganic nitrogen was added (1= NO_3_^-^ and NH_4_^+^, 2 = NO_3_^-^ only and 3 = NH_4_^+^ only). In treatment B amino acids were added in a millimolar concentration range. In treatment C amino acids were added in a micromolar concentration range. The medium NBglu was included as a control

Medium	DAPG* µg mL^-1^	Generation time (h)	Optical density* * * * (620) nm	Protein* * * * µg mL^-1^
NBglu	11.0±3.1	6.1	1.54±0.02a^1^	2926±223.8a
A1	ND* *	23.5	0.09±0.004b	73.9±25.5b
A2	ND	23.4	0.09±0.003b	73.9±13.4b
A3	ND	24.4	0.09±0.001b	66.9±23.7b
B1	BD* * *	5.2	1.03±0.052c	1421.1±125.4c
B2	BD	5.3	1.07±0.052c	1998.9±151.9c
B3	19.8±4.5	5.2	0.95±0.117c	1607.22±151.2d
C1	ND	25.3	0.09±0.01b	66.8±14.2b
C2	ND	42.1	0.09±0.007b	55.0±14.1b
C3	ND	25.4	0.11±0.004b	74.2±2.2b

*  after 24 hours of growth * *  ND not detected * * * BD below detection limit * * * *  after 72 hours of growth ^1^ Figures within columns followed by different letters are significantly different (P<0.05).
